# Metabolomic approach to profile functional and metabolic changes in heart failure

**DOI:** 10.1186/s12967-015-0661-3

**Published:** 2015-09-12

**Authors:** Martino Deidda, Cristina Piras, Christian Cadeddu Dessalvi, Emanuela Locci, Luigi Barberini, Federica Torri, Federica Ascedu, Luigi Atzori, Giuseppe Mercuro

**Affiliations:** Department of Medical Sciences “M. Aresu”, University of Cagliari, Asse didattico Medicina, SS Sestu KM 0.700, 09042 Monserrato, Italy; Department of Biomedical Sciences, University of Cagliari, Monserrato, Italy; Department of Public Health, Clinical and Molecular Medicine, University of Cagliari, Monserrato, Italy

**Keywords:** Heart failure, Metabolomics, Energy metabolism, Echocardiography, Brain natriuretic peptide

## Abstract

**Background:**

Heart failure (HF) is characterized by a series of adaptive changes in energy metabolism. The use of metabolomics enables the parallel assessment of a wide range of metabolites. In this study, we appraised whether metabolic changes correlate with HF severity, assessed as an impairment of functional contractility, and attempted to interpret the role of metabolic changes in determining systolic dysfunction.

**Methods:**

A 500 MHz proton nuclear magnetic resonance (^1^H-NMR)-based analysis was performed on blood samples from three groups of individuals: 9 control subjects (Group A), 9 HF patients with mild to moderate impairment of left ventricle ejection fraction (LVEF: 41.9 ± 4.0 %; Group B), and 15 HF patients with severe LVEF impairment (25.3 ± 10.3 %; Group C). In order to create a descriptive model of HF, a supervised orthogonal projection on latent structures discriminant analysis (OPLS-DA) was applied using speckle tracking-derived longitudinal strain rate as the Y-variable in the multivariate analysis.

**Results:**

OPLS-DA identified three metabolic clusters related to the studied groups achieving good values for R^2^ [R^2^(X) = 0.64; R^2^(Y) = 0.59] and Q^2^ (0.39). The most important metabolites implicated in the clustering were 2-hydroxybutyrate, glycine, methylmalonate, and myo-inositol.

**Conclusions:**

The results demonstrate the suitability of metabolomics in combination with functional evaluation techniques in HF staging. This innovative tool should facilitate investigation of perturbed metabolic pathways in HF and their correlation with the impairment of myocardial function.

**Electronic supplementary material:**

The online version of this article (doi:10.1186/s12967-015-0661-3) contains supplementary material, which is available to authorized users.

## Background

Heart failure (HF) is one of the most common chronic diseases in the USA and Europe [1]. Approximately 1–2 % of the adult population in developed countries suffers from HF [2] and, despite the availability of innovative therapies, it continues to be associated with an annual mortality rate of 10 %.

A growing body of evidence highlights the potential involvement of a decrease in cardiac energy metabolism in the pathogenesis and progression of HF [3]. During this metabolic remodeling, changes in substrate utilization, oxidative phosphorylation, and high-energy phosphate metabolism occur. Cardiac energy metabolism can be fully evaluated in animal models or in myocardial specimens obtained by biopsy or from explanted hearts, but human evaluations in vivo are limited to the assessment of glucose and fatty acid turnover rates [4], oxidative phosphorylation [5], and ATP transfer by PET or NMR [6].

Metabolomics (MBS) is the study of the complete profile of small-molecule metabolites in an organism and may provide a metabolic overview, not only resulting from changes in the expression of genes and RNA, but also as a result of protein activity and environmental factors, including nutrition and drug therapies [7, 8]. MBS has been shown to have a substantial impact on the investigation of various cardiovascular diseases [7, 8] and the number of studies on its application for HF assessment is growing [9–11], however, sometimes with conflicting results [9, 10]. Recently, mass spectrometry-based profiling of plasma metabolites was performed in over 400 HF patients by Cheng et al. in order to assess the diagnostic and prognostic value of MBS in HF. Their results showed that MBS is able to provide significant prognostic value, independent of brain natriuretic peptide (BNP) and other traditional risk factors [11].

In our study, a myocardial contractility parameter derived from echocardiography was used to build a metabolomic model of HF in which the metabolic variables are associated to systolic function, in order to better identify metabolites linked to cardiac function rather than to the general metabolic status. Our aims were to verify the ability of this approach to discriminate HF patients from healthy subjects, and also to identify individuals with different degrees of myocardial dysfunction on the basis of their specific metabolic profiles.

## Methods

### Study design and population

The study was approved by the Institutional Ethics Committee (Azienda Ospedaliero-Universitaria, University of Cagliari) and was performed in accordance with the Declaration of Helsinki. Enrolled subjects were informed of the purpose and methodology of the study and their written consent was obtained prior to inclusion.

The study population included patients with HF (Table 1) who were consecutively admitted to our division, and control subjects matched for sex, age, and body mass index, randomly selected from patients attending the departmental Outpatient Clinic (Table 1). The inclusion criterion was presence of HF, diagnosed in accordance with the European Society of Cardiology’s HF Guidelines [2]. Subjects with cachectic disease, non-congestive hepatic or renal dysfunction, heritable metabolic disorders, or those who had previously undergone metabolic therapy were excluded from the study. The exclusion criterion for controls was the presence of more than one cardiovascular risk factor (overweight, high low-density lipoprotein and triglycerides, diabetes, hypertension, or habitual smoking).

**Table 1:** Anthropometric and clinical data of the study population

	Group A (N = 9)	Group B (N = 9)	Group C (N = 15)
Age (years)	64.9 ± 8.3	66.1 ± 7.9	66.7 ± 9.9
M/F	7/2	8/2	10/5
Height (m)	1.63 ± 0.06	1.67 ± 0.51	1.67 ± 0.45
Weight (kg)	67.2 ± 12.4	74.0 ± 10.6	72.9 ±9.6
BMI (kg/m^2^)	25.2 ± 3.2	26.4 ± 3.9	26.04 ± 2.7
BSA (m^2^)	1.74 ± 0.17	1.85 ± 0.20	1.84 ± 0.26
Diabetes	1	1	1
NYHA class			
I	9	8	0
II	0	1	2
III	0	0	13*
IV	0	0	0
Aetiology			
Ischaemic	–	4	7
Valvular	–	1	1
DCM	–	4	6
Hypertensive	–	0	1
Other	–	0	0
Drugs			
β-blockers		9	14
ACE-inhibitors	–	6	9
ARBs	–	3	6
Diuretics	–	7	15
Aldosterone antagonists	–	3	5
Ca-antagonists	–	4	1
Antiarrythmics	–	1	2
Acetylsalicylic acid	–	6	10
Antiplatelet agents	–	0	1
Anticoagulants	–	1	2
Statins	–	7	9
Other hypolipidemic agents	–	0	3
Insulin	–	1	1
Oral antidiabetic agents	–	1	1
Digoxin	–	1	1
Nitroderivates	–	2	0
Other antihypertensive drugs	–	1	0

*BMI* body mass index, *BSA* body surface area, *DCM* dilated cardiomyopathy

* p < 0.02 vs Group B

The study population consisted of three groups: 9 controls (Group A), 9 HF patients with mild-moderate impairment of left ventricle ejection fraction (LVEF 35–50 %; Group B), and 15 HF patients with severe LVEF impairment (LVEF < 35 %; Group C). Patients and controls underwent a full cardiovascular assessment, including medical history evaluation, physical examination, blood pressure measurement, 12-lead electrocardiogram (ECG), and echocardiographic analysis. In addition, two blood samples were obtained by venipuncture of the antecubital vein (4-mL Vacuette with EDTA and 10-mL Li-Heparin Vacuette, for BNP and MBS analysis, respectively).

### BNP

Plasma concentrations of BNP were measured using a non-competitive immunofluorimetric test with high specificity (Triage^®^ BNP Test, Biosite Inc., San Diego, CA, USA; normal values <100 pg/mL).

### Conventional echocardiography and TDI and ST imaging

Echocardiographic assessments were conducted using a commercial system equipped with tissue Doppler imaging (TDI) and speckle tracking (ST) echocardiography (Toshiba Artida-Toshiba Corp., Tochigi, Japan). At least three sets of loops, consisting of three consecutive cardiac cycles, were stored for offline analysis. LVEF was measured using the modified Simpson’s biplane method from the apical 4- and 2-chamber view. Early filling (E wave) and atrial (A wave) peak velocities and E/A ratios were measured from transmitral flow. Using TDI, peak systolic (S), early diastolic (E′), and late diastolic (A′) mitral annular velocities were measured. Moreover, global longitudinal strain (GLS) and strain rate (SR) were evaluated using ST echocardiography.

### MBS analysis

Heparinized blood samples were immediately centrifuged at 4000 rpm for 15 min. Then, the supernatant was divided into aliquots and stored at −80 °C.

### Plasma chloroform/methanol/water extraction for NMR

All plasma samples were thawed and centrifuged at 12,000 rpm for 10 min. The supernatants were processed using chloroform/methanol/water extraction. Specifically, 800 μL of plasma was processed with 2.4 mL of chloroform/methanol (1:1, v/v) and 350 μL of H_2_O, vortexed for 30 s, and centrifuged at 4500 rpm for 30 min. After centrifugation, the hydrophilic and lypophilic fractions were collected. Approximately 1 mL of the hydrophilic fraction was dried overnight using a speed vacuum concentrator (Eppendorf) and stored at −80 °C until NMR analysis. ^1^H-NMR acquisition parameters and further data processing details are reported in the Additional file 1: Supporting Information.

### Multivariate statistical analysis

The multivariate statistical methods employed were: (1) the unsupervised principal components analysis (PCA) for sample distribution overview, (2) projection to latent structures by partial least squares (PLS) regression, and (3) orthogonal partial least square discriminant analysis (OPLS-DA) for the identification of the most discriminant variables that characterize groups. PCA is a technique that transforms the variables in a dataset into a smaller number of new latent variables, known as principal components. Each new principal component represents a linear combination of original variables, enabling the generation of a compact description of the variation within a given dataset. The OPLS-DA model maximizes the covariance between the measured data of the X-variable (peak intensities in NMR spectra) and the response of the Y-variable (class assignment) within the groups. Useful parameters obtained from the OPLS-DA model were the variable influence on projection (VIP) scores and coefficients that describe the metabolite influence over all validated components. The model quality was evaluated on the corresponding partial least square discriminant analysis (PLS-DA) model using a 7-fold cross-validation and permutation test. The generated R^2^ and Q^2^ values described the predictive ability and the reliability of the fitting, respectively.

### Univariate statistical analysis

A one-way ANOVA (analysis of variance) with Fisher’s LSD test was performed on the anthropometric, clinical, and echocardiographic parameters of the three groups. Furthermore, the same test was applied to the NMR data, in order to assess which spectral regions, and therefore which metabolites, were mainly involved in each of the various groups. A *P* value of p < 0.05 was considered statistically significant.

The receiver operating-characteristic (ROC) curve was analyzed with sensitivity versus 1 − specificity, and the area under the curve (AUC) was calculated using the free software package ROCCET: ROC Curve Explorer and Tester. An AUC > 0.8 indicates a test with a good discrimination between controls and patients.

## Results

No appreciable differences were observed in any of the anthropometric parameters between the three groups (Table 1). In agreement with the study design, patients in group B were modestly symptomatic or virtually asymptomatic, showing a substantial hemodynamic balance. Patients in group C showed clinical signs of HF. Each group included one subject affected by type II diabetes mellitus. All patients in groups B and C received optimized standard therapy, in line with their functional class and relative international recommendations [2]. Additionally, no significant differences were found between the two groups in terms of pharmacological treatment (Table 1).

### BNP

**Fig. 1:**
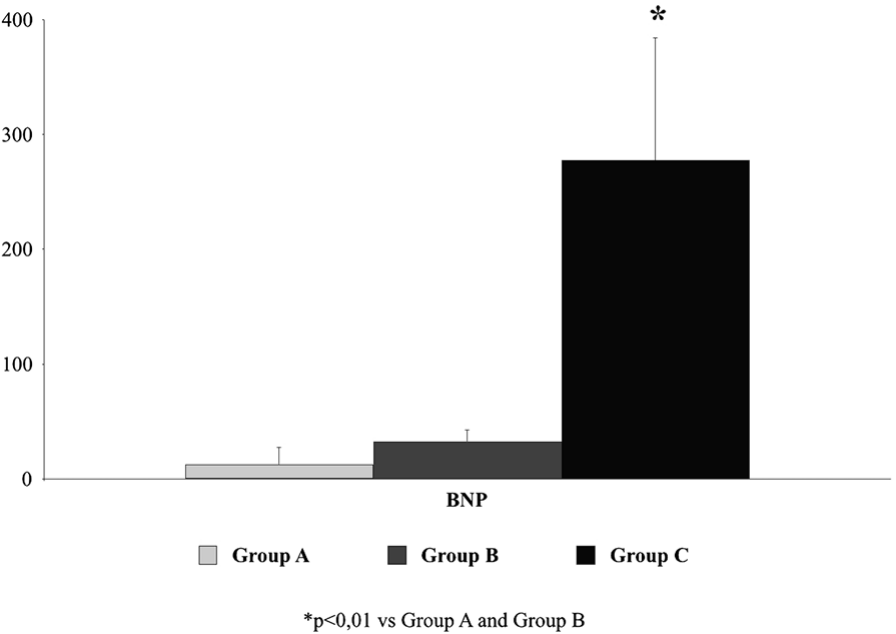
BNP values in the three groups

BNP values were in the normal range in groups A and B, but they were significantly increased in group C (p < 0.001; Fig. 1). The fact that healthy subjects in group A cannot be distinguished from those of group B on the basis of BNP is also noteworthy.

### Echocardiographic data

**Fig. 2:**
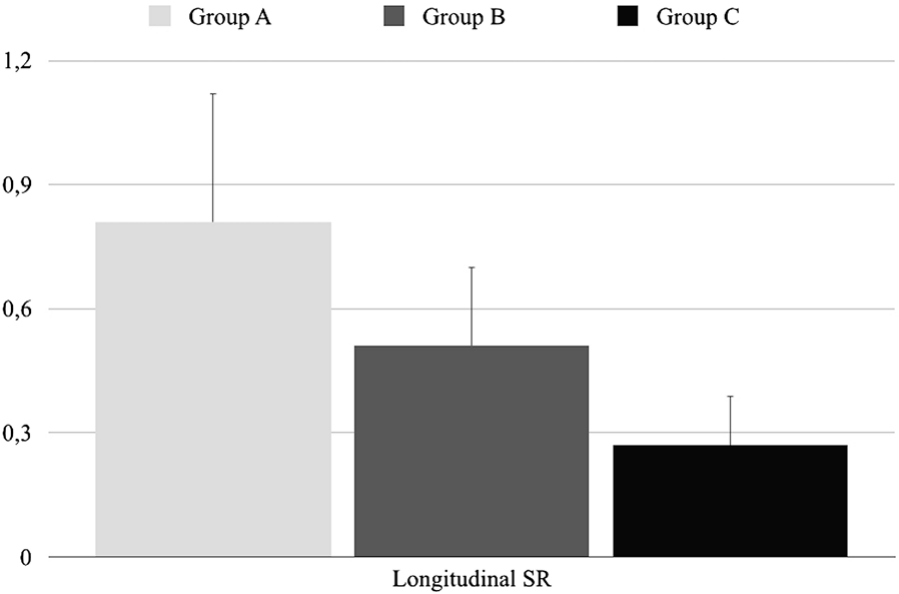
Longitudinal SR values in the three groups (one way ANOVA p < 0.0001)

LVEF featured normal values in group A, a mild to moderate impairment in group B vs. group A (p < 0.001), and a severe decrease in group C vs. group A and group B (p < 0.001). The progressive deterioration of LV function in the transition from group A to group B, and from the latter to group C, was confirmed by TDI and ST (Table 2; Fig. 2).

**Table 2:** Echocardiographic data in the three groups

	Group A	Group B	Group C
Systolic function			
LVEF (%)	61.3 ± 5.8	41.9 ± 4.0^##^	25.3 ± 10.3^##,∫∫^
S (cm/s)	7.63 ± 3.0	5.42 ± 1.91^#^	4.62 ± 1.84^##,∫^
GLS (%)	15.6 ± 3.2	9.9 ± 1.5^##^	4.9 ± 2.2^##,∫∫^
Diastolic function			
E/A	0.89 ± 0.30	0.74 ± 0.30	1.79 ± 1.70
E′/A′	0.78 ± 0.30	0.78 ± 0.38	0.79 ± 0.45
E/E′	9.83 ± 3.78	12.50 ± 5.95	21.07 ± 12.21*

*LVEF* left ventricle ejection fraction, *S* systolic peak velocity, *GLS* global longitudinal strain, *E* early ventricular filling peak velocity, *A* atrial (late) peak velocity, *E′* early mitral annular velocity, *A*′ late mitral annular velocity

* p < 0.05 vs Group A; ^#^p < 0.01 vs Group A; ^##^p < 0.001 vs Group A; ^∫^p < 0.05 vs Group B; ^∫∫^p < 0.001 vs Group B

A statistically significant difference in E/E′ ratio was observed in group C vs. group A, while no difference was found between group B and either group A or C (Table 2). However, E/E′ revealed a trend of moderate diastolic dysfunction in group B.

Intraobserver variability was previously reported for our EchoLab [12].

### ^1^H-NMR

**Fig. 3:**
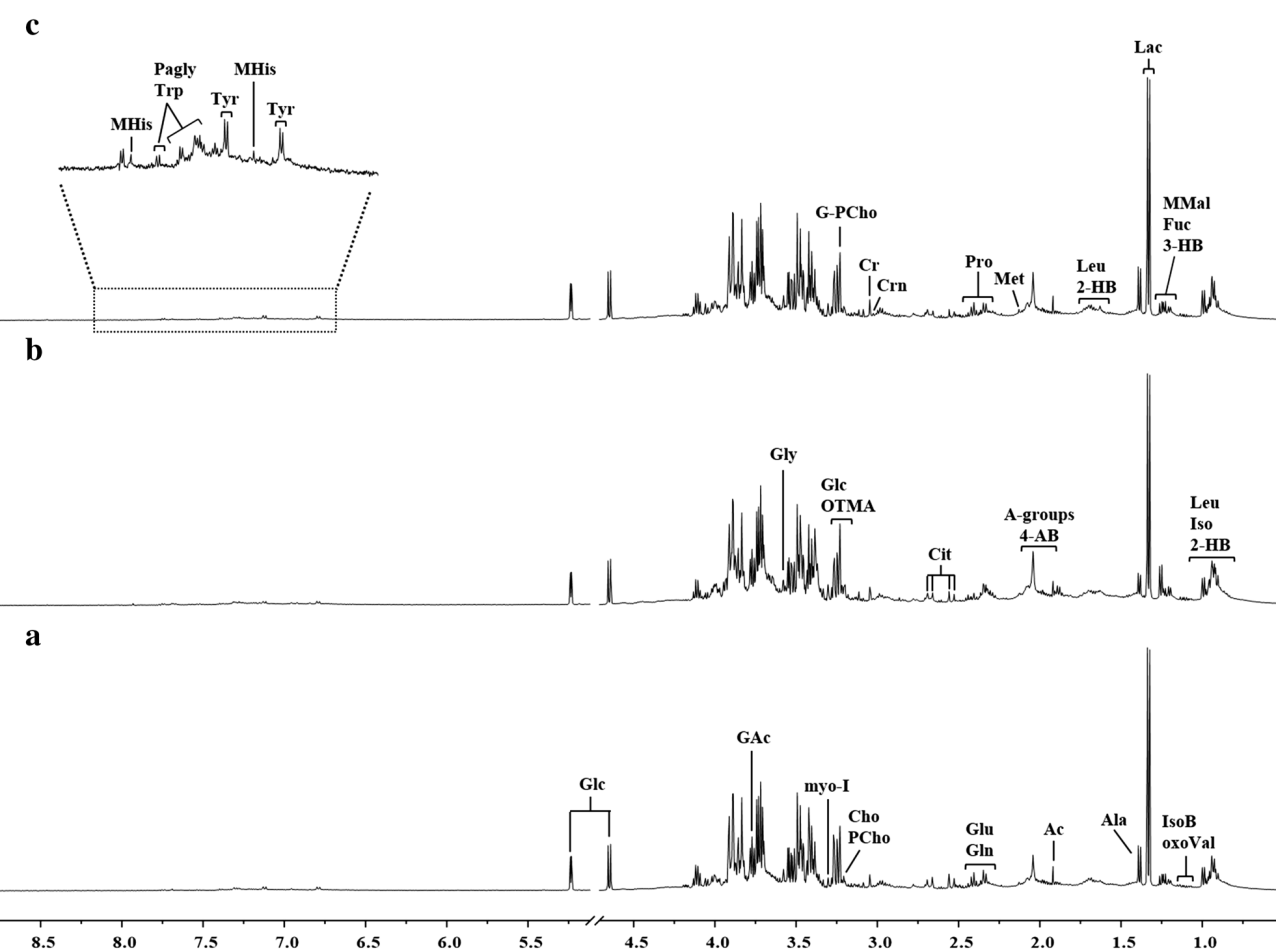
Spectral area assignments of a representative ^1^H NMR spectra of plasma obtained from **a** group A, **b** group B, and **c** group C. *Peaks* 2-hydroxybutyrate (2-HB), 3-hydroxybutyrate (3-HB), 3-methyl-2-oxovalerate (oxoVal), 4-aminobutyrate (4-AB), acetate (Ac), alanine (Ala), choline (Cho), citrate (Cit), creatine (Cr), creatinine (Crn), fucose (Fuc), glucose (Glc), glycine (gly), glycero-3-phosphocholine (G-PCho), glutamate (Glu), glutamine (Gln), guanidoacetate (GAc), isobutyrate (IsoB), isoleucine (Iso), lactate (Lac), leucine (Leu), methionine (Met), methylmalonate (MMal), methylhistidine (MHis), myo-inositol (myo-I), *N*-acetyl groups (A-groups), phenylacetylglycine (PAgly), phosphocholine (PCho), proline (Pro), trimethylamine *N*-oxide (OTMA), tryptophan (Trp), and tyrosine (Tyr)

The representative ^1^H-NMR spectra of plasma hydrophilic extracts of each of the three groups of samples are shown in Fig. 3. The spectral peaks were assigned to individual metabolites on the basis of literature research and using the 500 MHz library from Chenomx NMR suite 7.1 (Chenomx Inc., Edmonton, Alberta, Canada). Major peak assignments of plasma samples are illustrated in Fig. 3 and chemical shifts of all metabolites are summarized in Additional file 1: Table S1.

**Fig. 4:**
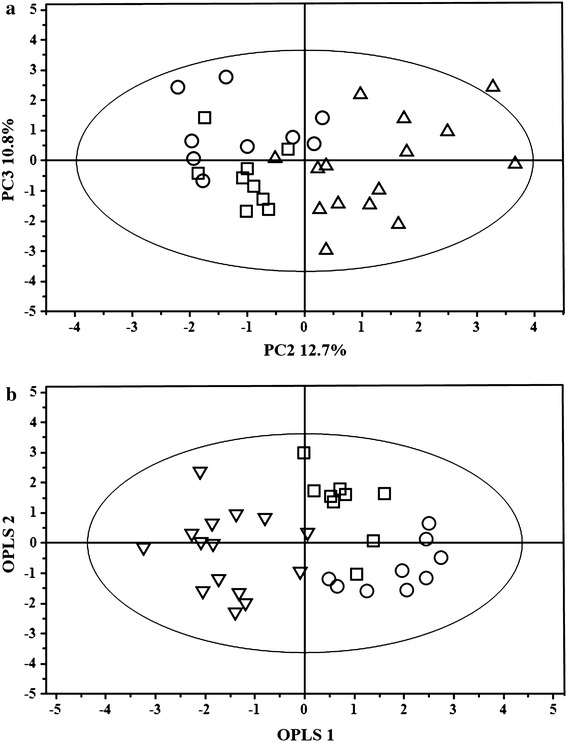
PCA scores (**a**) and OPLS-DA scores plots (**b**) of ^1^H NMR spectra of plasma samples: group A (*circle*), group B (*box*), and group C (*triangle*)

An unsupervised PCA was initially applied to the whole dataset to visualize possible metabolic differences among the groups, while at the same time identifying potential outliers. The first three PCs described 64.8 % of the variance, and all the samples were within the Hotelling’s T2 confidence ellipse. The PC2 vs. PC3 score plot (Fig. 4a) shows a clear tendency of samples to cluster on the basis of systolic function. In particular, along PC2, group C is separated form groups A and B, while these two are separated along PC3. These results clearly show that the three classes of samples have different metabolic profiles.

To investigate which metabolites significantly contribute to the observed separation, an OPLS-DA was applied to the spectral data. The score plot of the first and second predictive latent variable is shown in Fig. 4b. As illustrated, a good separation was observed between the three groups of samples. OPLS-DA modeling produced a model with three components, with R^2^(X) = 0.64, R^2^(Y) = 0.59, and Q^2^(Y) = 0.39, thereby indicating a good capacity for fitting and prediction. Additional information was obtained from the OPLS-DA model, through the study of the VIP scores and regression coefficients. In particular, VIP analysis allowed the identification of the metabolites contributing to the separation. The VIP value was used to reflect variable importance. Variables with a VIP score >1 were taken into consideration and the corresponding coefficient values for each class were studied to attribute discriminant metabolites to a specific group. The positive or negative value of the coefficient was used to determine upward or downward variation of the metabolite in the investigated spectral regions. The most significantly characterizing metabolites are reported in Table 3 and shown in Additional file 1: Fig. S1.

**Table 3:** Metabolites identified to have a VIP score greater than 1 through the OPLS-DA model of the three sample classes and corresponding regression coefficient values

Class	Variables	VIP	Coefficient values			Metabolites
			Group A	Group B	Group C	
Group A	1.94	2.46	0.14	−0.07	−0.06	Acetate
	0.9	2.40	0.07	0.05	−0.11	2-Hydroxybutyrate
	0.98	2.27	0.11	−0.02	−0.08	Leucine/isoleucine
	0.94	1.92	0.08	−0.01	−0.07	Leucine/isoleucine
	1.66	1.88	0.05	0.03	−0.08	2-Hydroxybutyrate/leucine
	1.18	1.67	0.09	−0.04	−0.04	3-Hydroxybutirate/ fucose
	1.46	1.52	0.06	−0.01	−0.05	Alanine/isoleucine
	1.5	1.44	0.04	0.03	−0.06	Alanine/isoleucine
	2.42	1.26	0.06	−0.06	0.01	3-Hydroxybutirate/glutamina
	1.1	1.22	0.05	0.01	−0.05	3-Methyl-2-oxovalerate
	1.7	1.20	0.04	−0.05	0.01	2-Hydroxybutyrate
	1.22	1.18	0.04	−0.05	0.01	3-Hydroxybutirate/ fucose
Group B	3.38	3.45	−0.22	0.13	0.08	Proline/methanol
	3.9	2.12	−0.08	0.07	0.01	Glucose
	3.22	1.78	−0.05	0.11	−0.06	Glycero-3-phosphocholine
	3.94	1.76	−0.06	0.07	−0.01	Glucose/creatine
	1.9	1.71	−0.07	0.11	−0.04	4-Aminobutyrate
	3.42	1.54	0.03	0.08	−0.10	Glucose
	3.74	1.45	−0.01	0.01	0.00	Glucose
	1.26	1.44	−0.04	0.09	−0.05	Methylmalonate
	1.86	1.25	−0.01	0.06	−0.05	4-Aminobutyrate
	2.34	1.17	−0.03	0.07	−0.04	3-Hydroxybutirate/glutamate
	3.98	1.07	−0.08	0.04	0.04	2-Hydroxybutyrate
	2.7	1.01	−0.07	0.05	0.02	Citrate
	1.34	4.43	0.00	−0.23	0.22	Lactate
	3.7	3.05	−0.15	0.01	0.12	Glucose
Group C	3.66	2.52	−0.12	−0.02	0.13	Glucose/glycero-3-phosphocholine
	3.62	2.50	−0.11	−0.03	0.13	Myo-inositol/glycero-3-phosphocholine
	3.58	2.40	−0.07	−0.05	0.11	Myo-inositol/glycine
	3.82	1.94	−0.03	−0.06	0.08	Glucose/guanidoacetate
	4.14	1.65	0.00	−0.09	0.08	Lactate
	3.3	1.51	−0.03	−0.05	0.07	Myo-inositol/glycine/OTMA
	4.02	1.48	−0.08	0.01	0.06	2-Hydroxybutyrate
	2.06	1.36	−0.06	−0.04	0.10	*N*-acetyl groups
	4.06	1.09	−0.05	−0.01	0.05	Myo-inositol/creatinine
	3.14	1.02	−0.08	0.03	0.04	Methylmalonate

Higher value of the coefficients indicates higher comparative level of the corresponding metabolite in the class

**Fig. 5:**
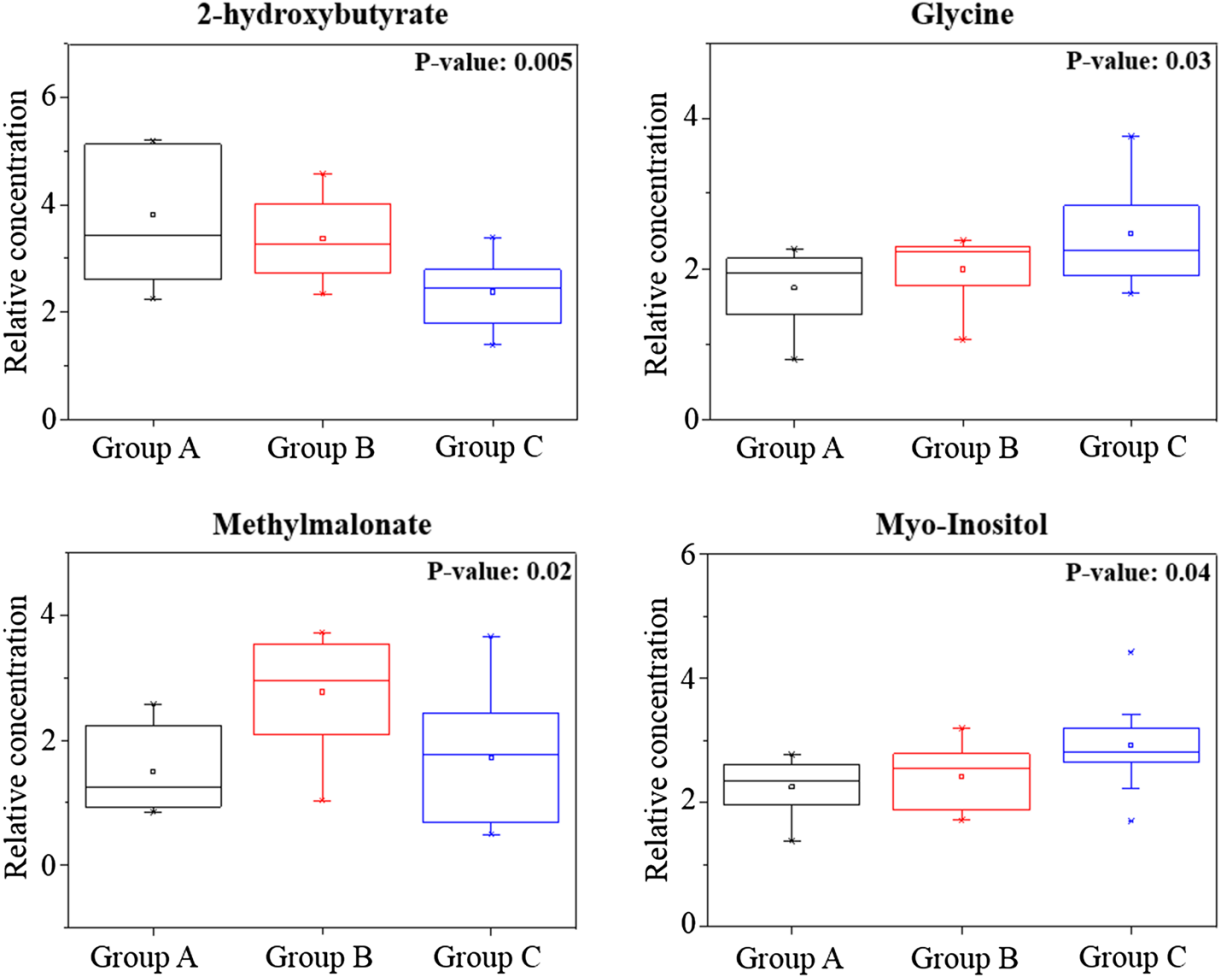
*Box-*and*-whisker* plots of the metabolite levels in patients with mild-moderate and severe HF (groups B and C groups) relative to healthy controls (group A). P values from one-way ANOVA with Fisher’s LSD test are displayed in the *upper right corner* of each plot

To understand the actual trend of the metabolites highlighted in Table 3, their relative concentrations were determined using Chenomx NMR and spectral regions were normalized and subjected to a one-way ANOVA test. Four metabolites (2-hydroxybutyrate, glycine, methylmalonate, and myo-inositol) showed significant variation. The performance of these metabolites was evaluated individually in the three groups with box-and-whisker plots (Fig. 5). Interestingly, group C exhibited a lower 2-hydroxybutirate content, but a higher content of glycine and myoinositol than groups A and B. Conversely, the methylmalonate content was higher in group B than in groups A and C. The combined performance of 2-hydroxybutyrate, glycine, and myo-inositol was evaluated using ROC curves. The levels of the three metabolites allowed to distinguish patients with severe HF, both from those with mild to moderate disease and from healthy controls with an AUC > 0.80 (Additional file 1: Fig. S2).

**Fig. 6:**
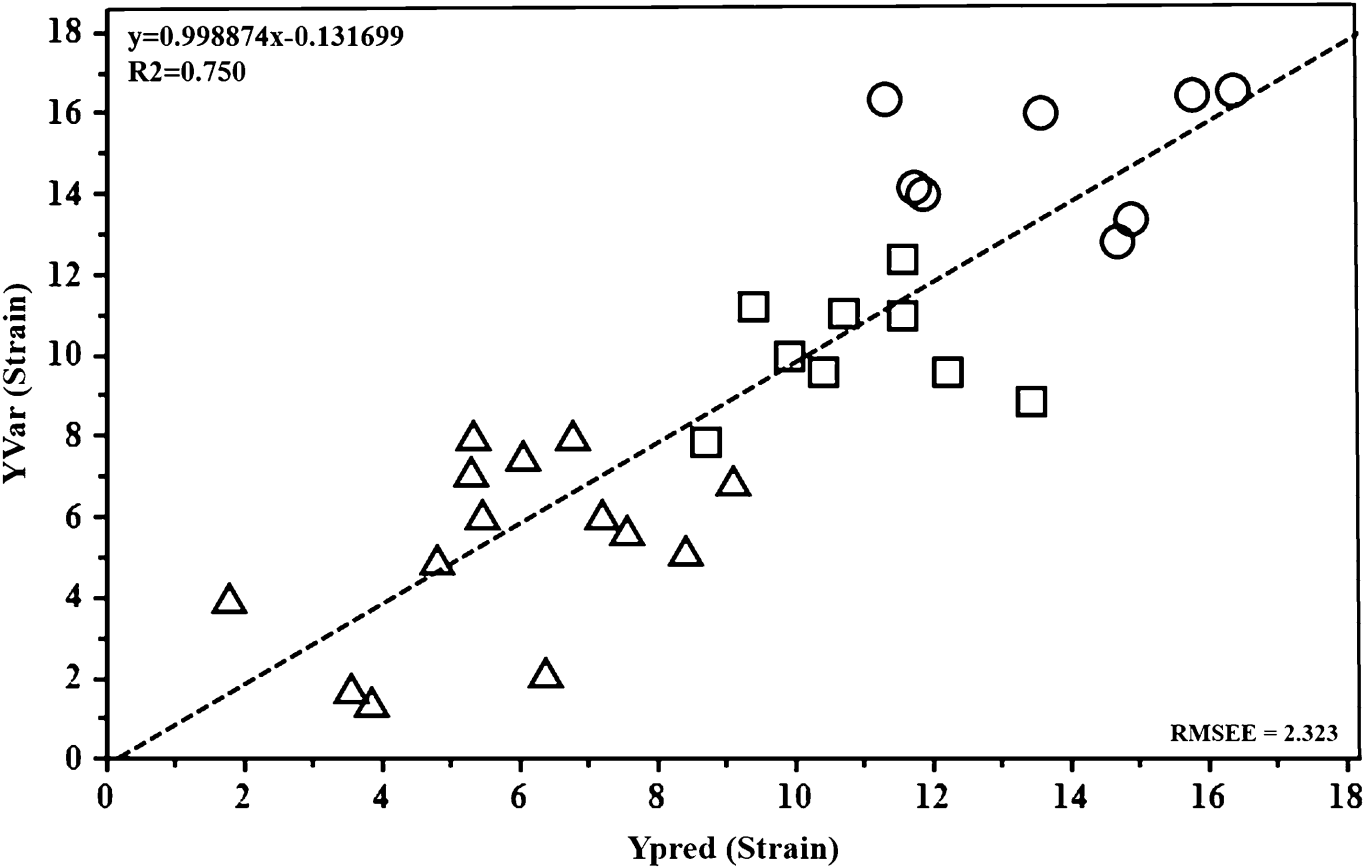
Predicted vs. measured longitudinal SR from PLS regression with NMR metabolic profiles of the three groups [group A (*circle*), group B (*box*) and group C (*triangle*)] as X-variables and longitudinal SR as the Y-variable

Finally, a PLS regression analysis was carried out in order to evaluate the relationship between different metabolic profiles and longitudinal SR (Fig. 6). The good overall correlation resulting from PLS regression [R^2^ = 0.750, and the R^2^(Y) and Q^2^(Y) values of 0.746 and 0.58, respectively] indicates the efficiency of the regression fitting and predictability of the resulting PLS mode. The permutation test, used to evaluate the statistical significance of the estimated predictive power of the model, shows an R^2^(Y) intercept of 0.293 and a Q^2^(Y) intercept of −0.222, thus confirming the validity of the PLS model.

## Discussion

This study focused on the MBS investigation of two groups of HF patients with mild-to-moderate and severe impairment of systolic function. The two groups of patients were compared with a control group using longitudinal SR as a landmark continuous variable of systolic function in order to try to trace metabolic alterations linked to myocardial contractility. The results show that: (a) MBS can identify a metabolic fingerprint specific for each HF stage, independent of BNP levels, which were similar in controls and patients with mild-to-moderate HF, and (b) different metabolic profiles are related to contractile function, as determined by longitudinal SR.

Cardiac metabolic abnormalities in HF may reflect both an increase in energy demand, and an impaired ability to generate a sufficient amount of ATP. Given that the heart is highly dependent on ATP, impairment of this process can rapidly induce contractile dysfunction [13, 14]. In order to recruit its residual contractile reserve, the failing heart consumes a disproportionate amount of energy, which is associated with worse clinical outcomes [15]. On this basis, longitudinal SR was used as an index of cardiac contractility and was correlated with metabolic changes that occur in HF. Several studies have shown that this parameter deteriorates precociously in cardiac disease, prior to the onset of clinical symptoms and LVEF reduction [16]. In addition, longitudinal SR is predictive of death or hospitalization for HF and adds an additional value in predicting death from any cause beyond LVEF [17]. The use of this parameter as the Y-variable in a multivariate analysis enabled us to identify the fingerprints of two well-separated stages of HF, that are associated with compensated myocardial function and advanced cardiac failure, highlighting the different metabolic frameworks correlated with the corresponding clinical features, and with increasing functional and metabolic impairment.

The use of MBS in HF has been recently evaluated in several studies, sometimes with contrasting results. In 2013, Tenori investigated the metabolomic fingerprint of the disease and its relationship with the levels of N-terminal of the prohormone brain natriuretic peptide and New York Heart Association (NYHA) classes in patients with chronic HF. He concluded that MBS is able to identify a metabolic cluster of HF, which correlates with the presence of disease, irrespective of its severity [10]. On the other hand, a study similar to ours classified HF patients in three groups of progressive disease severity, each characterized by a specific metabolic fingerprint [9]. The authors used the level of myocardial energy expenditure (MEE) as an index of cardiac performance. However, MEE is derived from the stroke volume and is based on the assessment of global LV performance. Parameters derived from regional and global LV function are not very sensitive, because they are highly dependent on loading and are likely to produce abnormal results if obtained under unusual load conditions, as is the case in HF [18]. More recently, Cheng et al. performed a mass spectrometry-based profiling of plasma metabolites in over 400 HF patients and 114 control subjects in order to evaluate the diagnostic and prognostic value of metabolomics in HF. The metabolic fingerprint resulting from this critical study consisted of histidine, phenylalanine, spermidine, and phosphatidylcholine C34:4, and has been shown to have a diagnostic value comparable to that of BNP. In addition, using the combined endpoints of death or HF-related rehospitalization, a metabolic panel, including the asymmetric methylarginine/arginine ratio, butyrylcarnitine, spermidine, and the total amount of essential amino acids, revealed a prognostic value independent of traditional risk factors, and more robust than that of BNP [11].

BNP is a widely used biomarker for the diagnosis and management of patients with recognized HF and an extensive body of evidence supports its use [2]. It is therefore important to emphasize that, while the OPLS-DA model based on different metabolic profiles in our study showed a significant cluster among the three groups, BNP levels were unable to differentiate patients with mild-to-moderate HF (group B) from control subjects (group A). The normal values of BNP that we found in patients with mild-to-moderate LV impairment probably reflect the balanced clinical condition of these patients. These findings seem to be consistent with the results of Cheng et al., as stated above [11].

The OPLS-DA model identified a set of metabolites that were decisive in determining the clustering of data originating from our subjects: glycine, myo-inositol, 2-hydroxybutyrate, and methyl-malonate. Beyond the diagnostic and clinical implications, the identification of the metabolic parameters in this study can lead to a deeper understanding of the pathophysiology of HF. In this respect, the broad spectrum of metabolites and their particular combination in MTB fingerprints could promote a more accurate interpretation of the adaptive metabolic response of the failing heart.

In myocardial hypertrophy and chronic HF, there is a decline in total creatine (Cr), due to a mismatch between the need for ATP and its actual production. The loss of Cr is myocardial-specific and occurs approximately one order of magnitude faster than that of ATP. Cr is synthesized primarily in the liver and pancreas from the amino acids glycine and arginine [19]. In accordance with this finding, we observed an increase in the levels of glycine in HF patients compared to controls, as well as a greater presence of this amino acid in group B than in group A. In patients with end-stage HF, there was an elevated myocardial activity of arginine-glycine aminotransferase, which decreases during recovery, due to therapy with left ventricular assist devices. This suggests that a specific metabolic response to HF involves high rates of local Cr synthesis [20]. In addition to its role in Cr synthesis, intracellular glycine has a protective effect against reoxygenation injury in mitochondria of cardiomyocytes subjected to ischemia or Ca^2+^-stress under normoxia [21]. Moreover, a role of this metabolite has been suggested in preserving energy production in the mitochondria of cancer cells or myocytes during acute cellular stress [22].

Group C was also characterized by higher levels of myo-inositol, an essential component of the plasma membrane that acts as an intracellular second messenger [23]. A rapid turnover of myo-inositol and of its phosphate compounds was identified after adrenergic stimuli [24] and was associated with sarcoplasmic calcium overload and the development of cardiomyopathies in rats [25]. Although a recent report of Santulli showed that leaky ryanodine, but not inositol-trisphosphate (I3P), receptors/calcium channels are able to determine mitochondrial Ca^2+^ overload and dysfunction in HF [26], Go et al. previously has demonstrated a decrease in ryanodine receptors mRNA by 31 % in the left ventricle of failing hearts, while IP3 receptors and the relative amount of IP3 binding sites were increased by, respectively 123 % and approx. 40 % [27]. Therefore, the observed increase in the levels of myo-inositol could be part of a compensatory response in advanced stages of HF. On the other hand, the same IP3 receptors may play a role in cardiomyocyte apoptosis and, therefore, in the progression of HF [28, 29].

The significance of changes in circulating and intracardiac ketone bodies in HF is still under investigation. Alexander et al. found high serum levels of 2-hydroxybutyrate in dilated cardiomyopathy patients. On the contrary, we observed lower concentrations of this metabolite in group C than in the control group. Previous MBS studies showed that the extraction of cardiac ketone bodies from the plasma depends on their concentration in circulation [30, 31], rather than on the presence and degree of LV dysfunction [29]. These results were confirmed by Psychogios, who reported higher levels of 2-hydroxybutyrate in healthy subjects than in patients who underwent heart transplantation [32].

The last metabolite identified in the univariate analysis, methylmalonate, was higher in patients from group B than in the other two groups. Kang and Chung, in 2011 and in 2012, respectively, demonstrated the presence of high levels of methylmalonate in urine samples of HF patients. Methylmalonate is an intermediate of one of the anaplerotic pathways, thus helping to maintain the efficiency of the Krebs cycle. Changes in the Krebs cycle precede the deterioration of systolic function [33] and a correlation between efficiency of the citric acid cycle and systolic function was detected [34]. On the basis of our data, it is conceivable that in HF patients in hemodynamic equilibrium, the increase of methylmalonate is caused by the activation of the anaplerotic pathway, in an attempt to maintain the necessary production of chemical energy through the Krebs cycle. This ability could have helped to maintain the functional capacity and hemodynamic compensation of these patients.

Taken together, the findings of our study describe a unique metabolic profile, discriminating not only between HF patients and controls, but also between HF subjects with mild-to-moderate and severe systolic dysfunction, suggesting that a progressive depletion of energy reserves could be the cause of the development of cardiac impairment. Therefore, MBS associated with functional data, such as longitudinal SR, appears to accurately reflect the metabolic changes underlying myocardial dysfunction in HF. Our data suggest a boosted energetic metabolism in early/compensated HF states, and a depletion of metabolic capacity that leads to a progressive systolic impairment in more severe HF cases.

## Limitations of the study

The small number of samples included in this study represents a considerable limitation. However, the sensitivity of the technique, together with the complimentary approaches used (clinical, echocardiographic, and MBS) enabled us to construct a promising model.

## Conclusions

MBS, in association with more traditional cardiac parameters, could be a valuable method for performing a thorough examination of the pathophysiology of HF and the metabolic alterations that accompany its deterioration.

From a practical point of view, the characterization of a myocardial impairment that is still free of symptoms could enable a more careful monitoring of at-risk individuals, allowing the anticipation of systolic function impairment and/or the development of an episode of overt failure. Clinical applications could be various: (1) monitoring chronic HF patients, including those waiting for a heart transplant, as well as relatives of subjects affected by heritable cardiomyopathies, and (2) early detection of cardiac involvement during cardiotoxic treatments (e.g., anthracycline therapy).

In addition, recognition of the metabolic pathways involved in the progression of the disease may help to identify new therapeutic targets at the molecular level, which could in the future enable medical providers to stop or reverse the myocardial and systemic adaptations of cardiac insufficiency.

## Additional file

Additional file 1. Supporting information describes in details ^1^H-NMR acquisition parameters and data processing.

## Abbreviations

HF: heart failure
ATP: adenosine triphosphate
PET: positron emission tomography
NMR: nuclear magnetic resonance
MBS: metabolomics
RNA: ribonucleic acid
LVEF: left ventricle ejection fraction
BNP: brain natriuretic peptide
TDI: tissue Doppler imaging
ST: speckle tracking
GLS: global longitudinal strain
SR: (longitudinal) strain rate
^1^H-NMR: proton nuclear magnetic resonance
PC(A): principal components (analysis)
PLS: partial least squares regression
OPLS-DA: orthogonal partial least square discriminant analysis
VIP: variable influence on projection
PLS-DA: partial least square discriminant analysis
MEE: myocardial energy expenditure
LV: left ventricle
Cr: creatine
