# Supplementary material for: Metabolomic approach to profile functional and metabolic changes in heart failure
Source: J Transl Med. 2015 Sep 12;13:297. doi: 10.1186/s12967-015-0661-3 (PMC4567812; doi:10.1186/s12967-015-0661-3)
Supplement: Supplementary file 1 — Additional file 1. Supporting information describes in details 1H-NMR acquisition parameters and data processing. [file 12967_2015_661_MOESM1_ESM.doc]

**SUPPORTING INFORMATION**

**Metabolomic approach to functional and metabolic myocardial changes in heart failure**

Martino Deidda^a^, MD, PhD; Cristina Piras^c^, PhD; Christian Cadeddu Dessalvi^a^, MD, PhD, FESC; Emanuela Locci^c^, PhD; Luigi Barberini^b^, PhD; Federica Torri^a^, MD; Federica Ascedu^a^, MD; Luigi Atzori^c^, MD, PhD; Giuseppe Mercuro^a^, MD

^a^Department of Medical Sciences “M.Aresu”, ^b^Department of Public Health, Clinical and Molecular Medicine and ^c^Department of Biomedical Sciences, University of Cagliari, Monserrato, Italy

**METHODS**

**^1^H-NMR spectroscopy and data processing**.

NMR experiments were carried out using a Varian UNITY INOVA 500 spectrometer operating at 499.839 MHz for proton and equipped with a 5 mm double resonance probe (Agilent Technologies, CA, USA). At the moment of analysis, dried hydrophilic plasma extracts were re-dissolved with 650 μL of D2O (99,8%, Cambridge Isotope Laboratories Inc, Andover, USA) containing the internal standard sodium 3-trimethylsilyl-propionate-2,2,3,3,-*d_4_* (TSP, 98 atom % D, Sigma-Aldrich, Milan) at a 0.5 mM final concentration, and transferred into 5 mm o.d. NMR tubes. ^1^H NMR spectra were acquired at 300K with a spectral width of 6000 Hz, a 90° pulse, an acquisition time of 2 s, a relaxation delay of 2 s, and 256 scans. The residual water signal was suppressed by applying a presaturation technique with low power radiofrequency irradiation for 2 s. ^1^H NMR spectra were imported in MestReNova software (Version 7.1.2, Mestrelab Research S.L.) and pre-processed with line broadening of 0.5 Hz, zero-filled to 64K, and Fourier transformed. Each spectrum was phased and baseline corrected. Chemical shifts were referred to the TSP single resonance at 0.00 ppm. The ^1^H-NMR spectra were reduced into consecutive integrated spectral regions (bins) of equal width (0.04 ppm) corresponding to the region 0.50–8.66 ppm. The spectral region between 4.74 and 4.94 ppm was excluded from the analysis to remove the effect of variations in the presaturation of the residual water resonance. The integrated area within each bin was normalized to a constant sum of 100 for each spectrum in order to minimize the effects of variable concentration among different samples. The final data set consisted of a 33×199 matrix that was imported into the SIMCA-P+ program (Version 13.0, Umetrics, Sweden), mean-centered and Pareto scaled column wise.

**
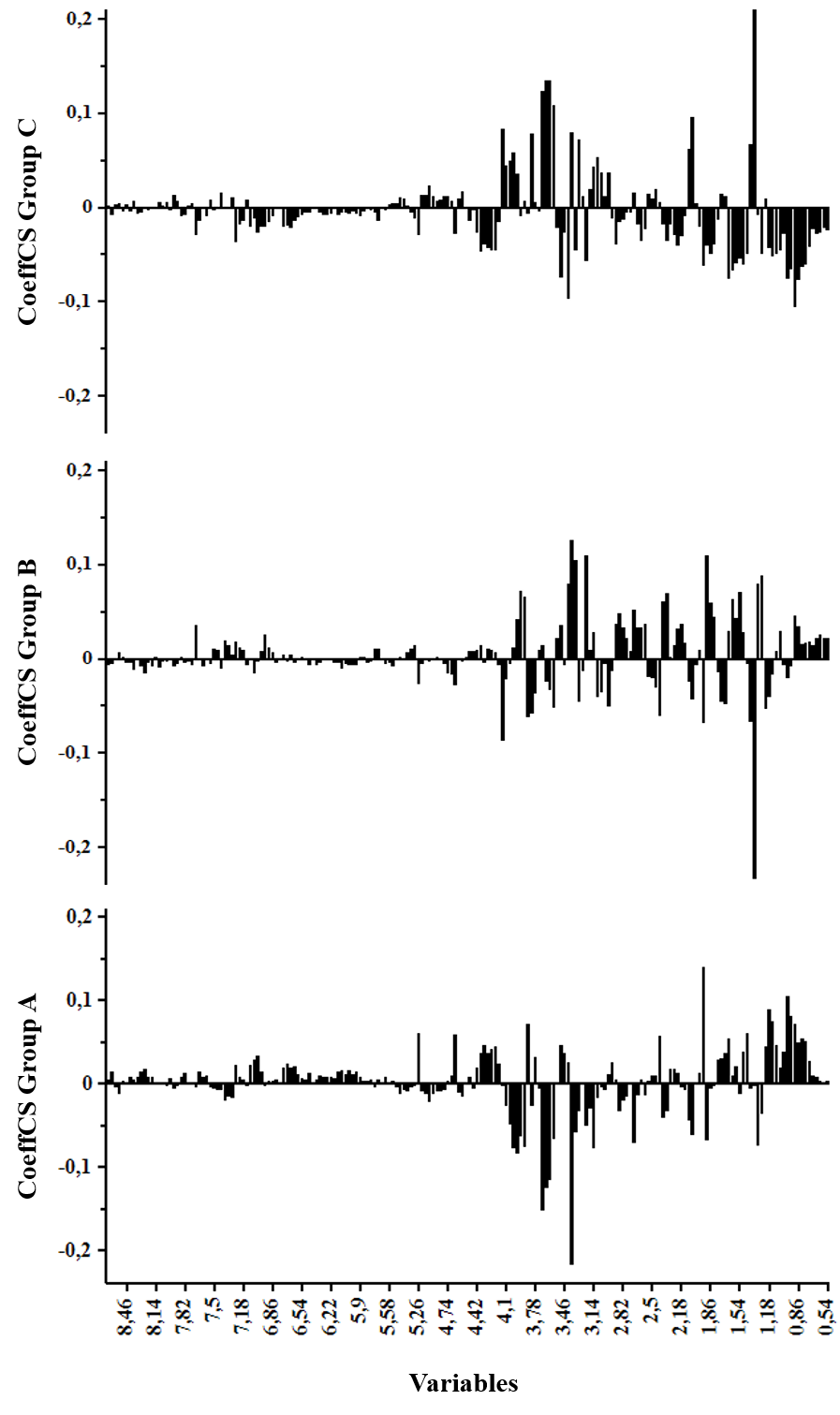
**

**Figure 1S**. OPLS-DA coefficient loading plots of plasma metabolites derived from healthy controls (A group) and heart failure patients mild-moderate and severe (B and C groups). Three groups have different spectral features, corresponding to a different distribution of metabolites. The positive or negative direction of a spectral regions (bins) represents increase or decline of the level of metabolite.


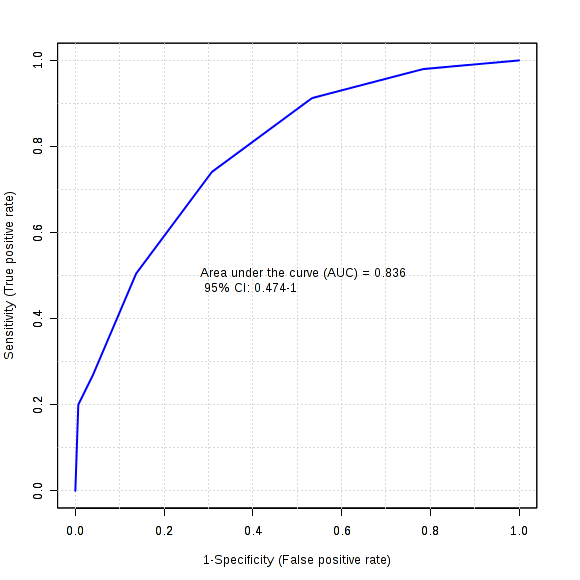


**Figure 2S.** Receiver Operating Characteristic (ROC) analysis for candidate plasma biomarkers. The area value under the ROC curve, shown in the middle of the picture, indicates the overall ability of a biomarkers to discriminate heart failure mild-moderate patients and healthy controls from severe heart failure patients.

**Table 1S.** Proton chemical shifts of the metabolites identiﬁed in the 500 MHz ^1^H spectra of plasma samples.

| **Metabolite** | **Group** | **^1^H ppm^a^** | **^1^H Multiplicity^b^** |
| --- | --- | --- | --- |
| 2-hydroxybutyrate | CH_3_ | 0.90 | t |
|  | CH_2_ | 1.68 | m |
|  | CH | 4.02 | dd |
| 3-hydroxyburyrate | CH_3_ | 1.20 | d |
|  | CH_2_ | 2.35 | dd |
|  | CH | 4.12 | m |
| 3-methyl-2-oxovalerate | CH_3_ | 0.88 | t |
|  | CH_3_ | 1.1 | d |
| 4-aminobutyrate | CH_2_ | 0.87 | m |
|  | CH_2_ | 2.29 | t |
|  | CH_2_ | 2.99 | t |
| Acetate | CH_3_ | 1.91 | s |
| Alanine | CH_3_ | 1.47 | d |
|  | CH | 3.78 | q |
| Choline/Phosphocholine | N-(CH_3_)_3_ | 3.19 | s |
|  | αCH_2_ | 3.51 | m |
|  | βCH_2_ | 4.05 | m |
| Citrate | γCH_2_ | 2.55 | d |
|  | γ′CH_2_ | 2.68 | d |
| Creatine | N-CH_3_ | 3.03 | s |
|  | N-CH_2_ | 3.93 | s |
| Creatinine | N-CH_3_ | 3.04 | s |
|  | N-CH_2_ | 4.06 | s |
| Fucose | αCH_3_ | 1.20 | d |
|  | βCH_3_ | 1.23 | d |
| Glycine | αCH_2_ | 3.57 | s |
| α-Glucose | C1H | 5.23 | d |
|  | C2H | 3.54 | dd |
|  | C3H | 3.70 | dd |
|  | C4H | 3.42 | dd |
| Table S1 (Continued) |  |  |  |
| **Metabolite** | **Group** | **^1^H ppm^a^** | **^1^H Multiplicity^b^** |
|  | C5H | 3.83 | m |
|  | C6H | 3.78 | m |
| β-Glucose | C1H | 4.64 | d |
|  | C2H | 3.24 | dd |
|  | C3H | 3.49 | t |
|  | C4H | 3.41 | dd |
|  | C5H | 3.46 | m |
|  | C6H | 3.90 | dd |
| Glutamate | αCH | 3.75 | m |
|  | βCH_2_ | 2.12 | m |
|  | γCH_2_ | 2.34 | m |
| Glutamina | αCH | 3.77 | m |
|  | βCH_2_ | 2.14 | m |
|  | γCH_2_ | 2.43 | m |
| Guanidoacetate | CH_2_ | 3.80 | s |
| Isobutyrate | CH_3_ | 1.12 | d |
| Isoleucine | αCH | 3.66 | m |
|  | βCH | 1.98 | m |
|  | γCH | 1.46 | m |
|  | γ'CH | 1.25 | m |
|  | γ'CH_3_ | 0.99 | d |
|  | δCH_3_ | 0.93 | t |
| Lactate | βCH_3_ | 1.32 | d |
|  | αCH | 4.10 | q |
|  | εCH_2_ | 3.02 | t |
| Leucine | αCH | 3.73 | t |
|  | βCH_2_ | 1.72 | m |
|  | γCH | 1.76 | m |
|  | δCH_3_ | 0.94 | d |
| Table S1 (Continued) |  |  |  |
| **Metabolite** | **Group** | **^1^H ppm^a^** | **^1^H Multiplicity^b^** |
| Methionine | αCH | 3.85 | t |
|  | βCH_2_ | 2.15 | m |
|  | γCH_2_ | 2.65 | t |
|  | S-CH_3_ | 2.13 | s |
| Methylmalonate | CH_3_ | 1.25 | d |
|  | CH | 3.16 | m |
| Methylhistine | βCH_2_ | 3.15 | dd |
|  | β′CH_2_ | 3.06 | dd |
|  | CH_3_ | 3.68 | s |
|  | C2H, ring | 7.03 | s |
|  | C4H, ring | 3.73 | s |
| Myo-Inositol | C1,3H | 3.54 | dd |
|  | C2H | 4.06 | t |
|  | C5H | 3.28 | t |
|  | C4,6H | 3.62 | t |
| N-Acetyl groups | CH_3_ | 2.06 | s |
| Phenylacetylglycine | CH_2_ | 3.66 | s |
|  | N-CH_2_ | 3.73 | d |
|  | C2,6H, ring | 7.35 | m |
|  | C3,5H, ring | 7.36 | m |
|  | C4H, ring | 7.42 | m |
| Proline | αCH | 4.13 | dd |
|  | βCH_2_ | 2.03 | m |
|  | β′CH_2_ | 2.36 | m |
|  | γCH_2_ | 1.99 | m |
|  | δCH_2_ | 3.34 | m |
|  | δ′CH_2_ | 3.42 | m |
|  |  |  |  |
|  |  |  |  |
| Table S1 (Continued) |  |  |  |
| **Metabolite** | **Group** | **^1^H ppm^a^** | **^1^H Multiplicity^b^** |
| Sn-Glycero-3-Phosphocholine | N-(CH_3_)_3_ | 3.22 | s |
|  | CH_2_ | 3.64 | m |
|  | O-CH_2_ | 3.90 | m |
|  | P-O-CH_2_ | 4.30 | m |
| Trimethylamine N-oxide | CH_3_ | 3.26 | s |
| Tryptophan | C4H, ring | 7.71 | d |
|  | C7H, ring | 7.53 | d |
| Tyrosine | C2,6H, ring | 6.86 | d |
|  | C3,5H, ring | 7.18 | d |

**^a^** ^1^H chemical shifts re reported with respect to TSP signal (0.00 ppm).

**^b^** Multiplicity definitions: s, singlet; d, doublet; t, triplet; q, quartet; dd, doublet of doublets; m, multiplet.
